# DFSP: A fast and automatic distance field-based stem-leaf segmentation pipeline for point cloud of maize shoot

**DOI:** 10.3389/fpls.2023.1109314

**Published:** 2023-01-31

**Authors:** Dabao Wang, Zhi Song, Teng Miao, Chao Zhu, Xin Yang, Tao Yang, Yuncheng Zhou, Hanbing Den, Tongyu Xu

**Affiliations:** ^1^ College of Information and Electrical Engineering, Shenyang Agricultural University, Shenyang, China; ^2^ College of Science, Shenyang Agricultural University, Shenyang, China; ^3^ School of Mathematics and Computer Science, Zhejiang Agriculture and Forestry University, Hangzhou, China; ^4^ School of Information and Intelligence Engineering, University of Sanya, Sanya, China

**Keywords:** point cloud, segmentation, maize, distance field, quickshift++

## Abstract

The 3D point cloud data are used to analyze plant morphological structure. Organ segmentation of a single plant can be directly used to determine the accuracy and reliability of organ-level phenotypic estimation in a point-cloud study. However, it is difficult to achieve a high-precision, automatic, and fast plant point cloud segmentation. Besides, a few methods can easily integrate the global structural features and local morphological features of point clouds relatively at a reduced cost. In this paper, a distance field-based segmentation pipeline (DFSP) which could code the global spatial structure and local connection of a plant was developed to realize rapid organ location and segmentation. The terminal point clouds of different plant organs were first extracted *via* DFSP during the stem-leaf segmentation, followed by the identification of the low-end point cloud of maize stem based on the local geometric features. The regional growth was then combined to obtain a stem point cloud. Finally, the instance segmentation of the leaf point cloud was realized using DFSP. The segmentation method was tested on 420 maize and compared with the manually obtained ground truth. Notably, DFSP had an average processing time of 1.52 s for about 15,000 points of maize plant data. The mean precision, recall, and micro F1 score of the DFSP segmentation algorithm were 0.905, 0.899, and 0.902, respectively. These findings suggest that DFSP can accurately, rapidly, and automatically achieve maize stem-leaf segmentation tasks and could be effective in maize phenotype research. The source code can be found at https://github.com/syau-miao/DFSP.git.

## 1 Introduction

Maize is one of the most important food crops in the world. Therefore, its production is essential in ensuring global food supplies. High-throughput phenotypic measurement is crucial for future maize variety improvement. The 3D sensing technologies, such as 3D laser scanners (Rist et al., 2018), multi-view images (Wu et al., 2020), and lidar (Jin et al., 2021), have been recently used for plant phenotype parameter measurements based on 3D point clouds. The organ-level segmentation of plant point clouds is necessary if the measurements of the organ-level phenotype indicators, such as leaf length and width, are involved. Numerous studies have evaluated point cloud segmentation at the organ level.

Plant organs are mainly classified and segmented using the local geometric features of point clouds. Tensor based features are simple features formed by combining the eigenvalues of neighborhood points. However, these features can only be used for simple stem and leaf segmentation tasks (Elnashef et al., 2019). The Point Feature Histogram (Rusu and Cousins, 2011) has been widely used in the segmentation of grapes (Paulus et al., 2013; Wahabzada et al., 2015), sorghum (Vijayarangan et al., 2018), and tomato (Ziamtsov and Navlakha, 2019) through integration with machine learning, clustering, and regional growth methods. The traditional point cloud feature extraction methods should be improved to enhance the segmentation accuracy and reduce computational time in the extraction of neighborhood point cloud features for each point in the plant.

Geometric features of organs can also be used for segmentation. The stem segmentation step is crucial in organ segmentation process. Stem point cloud removal weakens the connection between the remaining organs, allowing clustering methods to achieve instant segmentation. Although early methods mostly used the cylindrical fitting strategy to identify stems (Paproki et al., 2012; Gelard et al., 2017), the methods relied on appropriate parameter selection and required high resolution and point cloud quality.

The global topological structure of plant point cloud is also widely used for organ segmentation. Currently, most studies use point cloud skeletons to describe the topology of plants and segment the stems and leaves according to the topological relationship. This method is extremely dependent on the quality of the extracted skeleton. Currently, slice-based (Xiang et al., 2019; Zermas et al., 2020) and Laplacian-based methods (Wu et al., 2019; Miao et al., 2021b) are commonly used to extract plant point cloud skeletons. However, the slice-based method requires that the point cloud is aligned with the coordinate axis, which limits its application. Meanwhile, Laplacian-based method does not need point cloud alignment, but it is often incomplete or wrong when applied to plants, resulting in reduced segmentation accuracy. As a result, certain interactive corrections are usually made to ensure the segmentation accuracy of the Laplacian-based method. Notably, the Laplacian-based method has low computational efficiency.

Numerous studies have recently used deep learning network data to abstract point cloud features for semantic and instance segmentation of the organs (Griffiths and Boehm, 2019; Jin et al., 2019; Li et al., 2022a; Li et al., 2022b; Li et al., 2022c). Deep learning can learn local geometric features and global structural features of plant point clouds from data. Although deep learning has high segmentation effectiveness and efficiency, it requires a lot of data for training. Manual labeling of these data is time-consuming, labor-intensive, and expensive. Moreover, the current deep learning technology requires a down-sampling of the point cloud to a very low resolution (4096 or less), leading to the loss of numerous potential geometric features of small organs and plants.

The success of deep learning proves that the combination of global features and local features of point clouds can improve segmentation accuracy. However, getting enough data for training models in many application scenarios is difficult. In this study, an unsupervised segmentation method, which could easily integrate the global and local features of the plant was developed to achieve fast and accurate stem and leaf segmentation of maize. Like most crops, maize plants have many branching structures. This global structure makes the end areas of organs (leaf tip and the lowest end of the stem) more dispersed in 3D space, facilitating the positioning of these areas. The local connection between maize organs is also fixed. These global and local spatial morphological structures can be used to improve the efficiency of plant point cloud segmentation. A distance field-based segmentation pipeline (DFSP) was developed to integrate these features. The Minkowski distance field of maize point cloud was constructed to code these structures. Quickshift++ can theoretically operate on the distance field to realize rapid organ location and segmentation. The maize stem and leaf segmentation method combined with DFSP obtained the following characteristics: 1) The method encoded the global morphological structure information of maize plants and the local connection relationship of organs; 2) The method could limit time-consuming geometric feature calculation to a few organ end point clouds, thus effectively integrating local features and enhancing the efficiency of organ semantic recognition; 3) This method could automatically find the organ position, thus automating the whole segmentation process; 4) The method could segment the leaves from the tip, and deal with a situation where multiple new leaves are close to each other, and larger new leaves surround smaller leaves. In summary, the method provides an effective unsupervised automatic segmentation scheme for maize stems and leaves, enhancing organ segmentation efficiency equivalent to that of a deep learning model without additional data training.

## 2 Materials and methods

**Figure 1:**
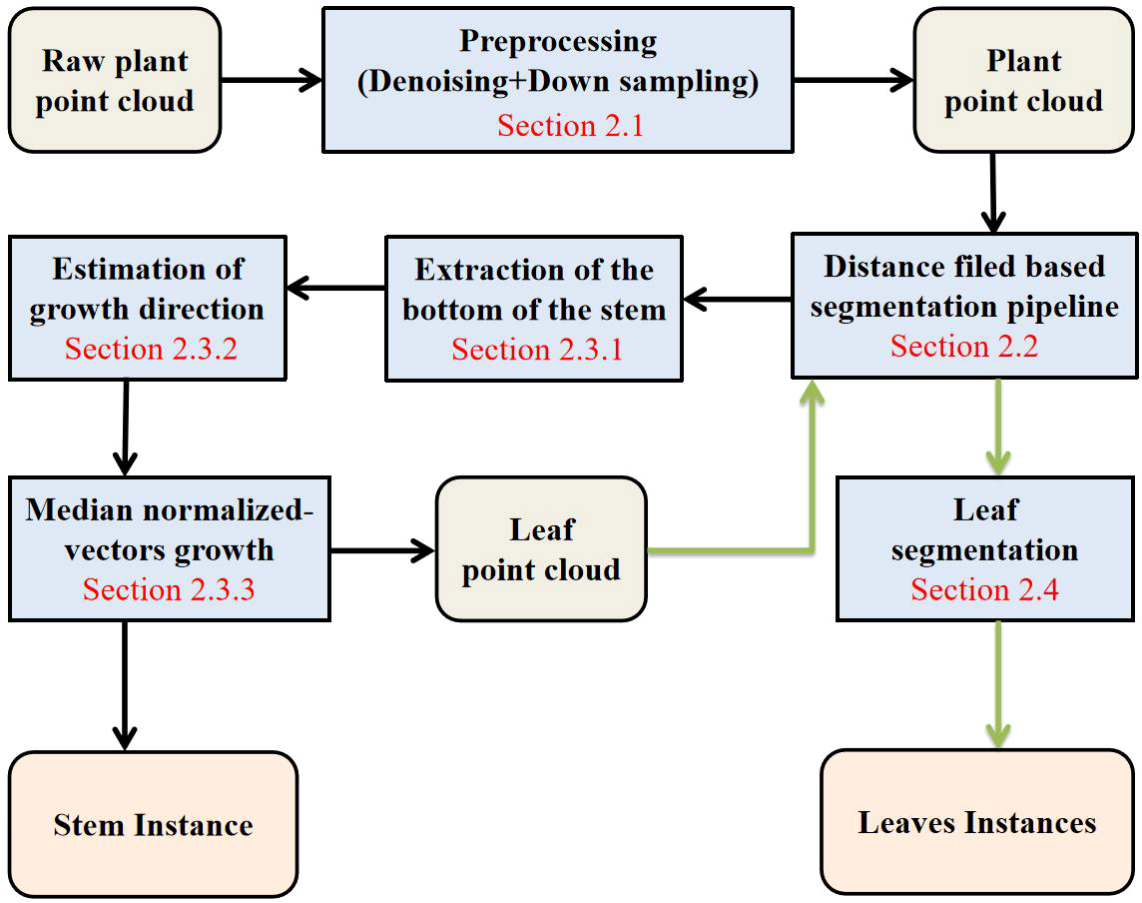
Flow chart of this article method.

This paper is organized into three main sections: the methodology, results, and discussion, excluding the introduction and abstract. The methodology introduces the data acquisition process and preprocessing method (2.1). Section 2.2 introduces the distance field-based segmentation pipeline (DFSP). The segmentation process was conducted for the subsequent stem and leaf segmentation tasks. Section 2.3 of the methodology introduces the stem extraction method. DFSP was used to automatically extract the low-end point cloud of the stem (2.3.1), quickly estimate the growth direction (2.3.2) on this basis and perform the median normalized-vectors growth segmentation (2.3.3). Section 2.4 outlines how the DFSP was used to segment the remaining leaf point cloud instances. The results of the study are presented in section 3 and then discussed in section 4. The flowchart of the entire process is shown in **Figure 1**.

### 2.1 Data acquisition and preprocessing

Field experiments were conducted in the experimental maize field of Shenyang Agricultural University (41.83 N, 123.56E) between May and July of 2019, 2020, and 2021. Five maize varieties (XianYu 335, LD145, LD502, LD586, and LD 1281) were planted in plots with an area of 666 m² with a row-row and plant-plant distances of 60 cm and 25 cm, respectively. Maize samples (420) were randomly chosen and transplanted into pots in an indoor laboratory. The raw point clouds were then obtained using a 3D laser scanner (FreeScan X3, Tianyuan Inc, Beijing, China). The raw data included the pot and other surrounding object point clouds. We used the CloudCompareStereo software to manually remove these points, leaving only the plant point cloud. The number of point cloud data from the different numbers of leaves in the various maize varieties is shown in **Table 1**. A more detailed description of the FreeScan X3 scanner and data acquisition environment is shown in **Table 2**.

**Table 1:** Number of plant samples from different numbers of leaves in the various maize varieties.

The number of leaves	Number of plant samples	XY335 data bulk	LD145 data bulk	LD502 data bulk	LD586 data bulk	LD1281 data bulk
3	17	17	0	0	0	0
4	39	32	3	0	1	3
5	100	87	2	3	4	4
6	100	86	4	3	5	2
7	60	46	3	3	4	4
8	36	33	1	0	1	1
9	28	22	1	4	1	0
10	24	13	3	4	3	1
11	10	5	1	2	0	2
12	6	2	0	0	2	1

**Table 2:** General specification of FreeScan laser scanner.

Specification	Value
Price	120000 yuan
Scanning range	280×250 mm
Scanning accuracy	0.030 mm
Working distance	300 mm
Depth of field	250 mm
Range	0.1-6 m (scalable)
Resolution	0.100 mm
Data acquisition time	All day
Data acquisition efficiency	7~15 min/plan
Environment	Indoor/Calm/Normal temperature/Artificial illumination

The pass-through and statistical outlier removal filters were used to denoise the point cloud and manually segment the original point cloud to obtain the ground truth. Too much data significantly reduces the segmentation efficiency when performing point-cloud segmentation. In this study, the segmentation process was improved using the method described by Miao et al. (2021a). The number of point clouds was first down-sampled to about 15,000 points while maintaining the local geometric characteristics of maize. The sampled point clouds were then segmented. Notably, the segmentation results of the subsampled point clouds could be up-sampled to the original point clouds through the sample-based segmentation method if the segmentation results in the original point cloud were needed.

**Figure 2:**
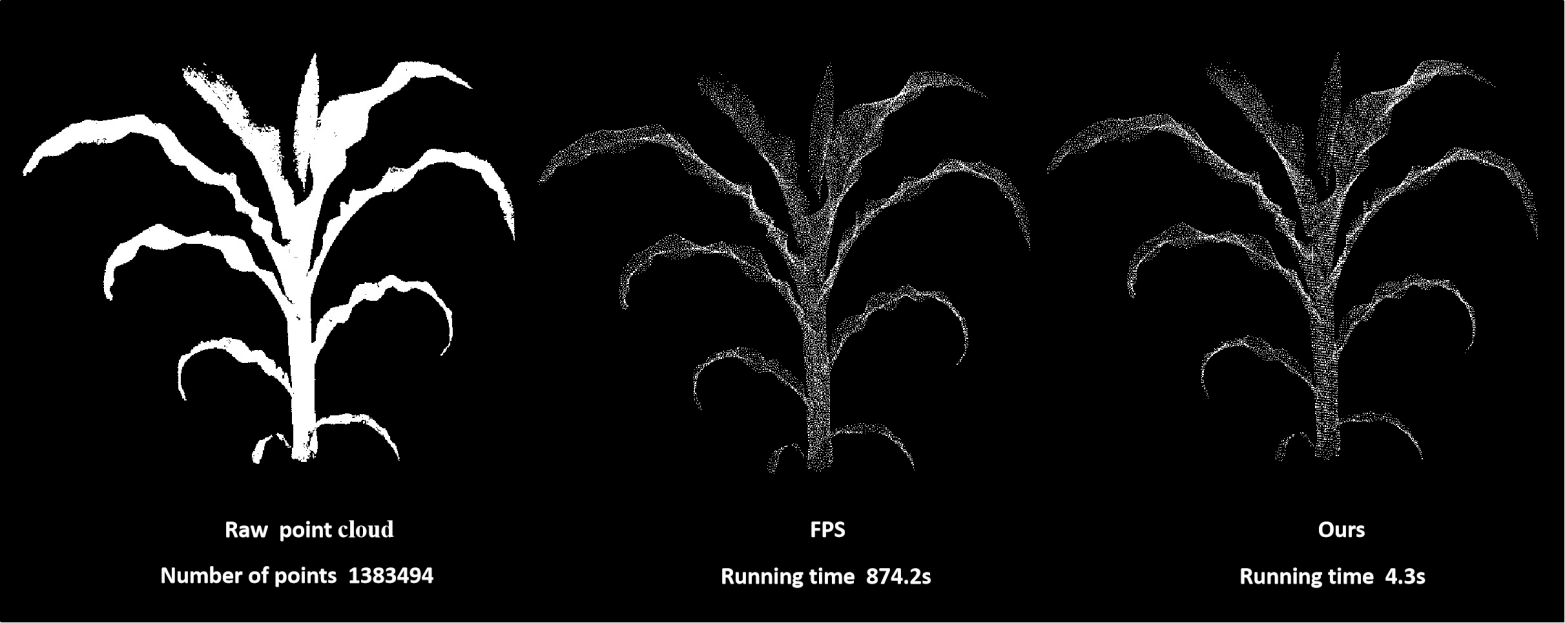
A comparison plot of plant point cloud down-sampling methods.

Besides maintaining the original geometry of the point cloud, the down-sampling process also reduced the number to a fixed 15,000 points. The fixed number of point clouds enables a better generalization of the parameter setting of the segmentation algorithm. Although farthest point sampling (FPS) is a suitable down-sampling method, it lasts longer because of the excessive number of original point clouds in the hundreds, thousands, and millions of levels. Moreover, the voxel-grid filter (Rusu and Cousins, 2011) was integrated with FPS to enhance efficiency. Although the voxel-grid filter is highly efficient and can maintain the original point cloud form, it does not guarantee the number of point clouds. As a result, the point clouds were down-sampled to slightly above 15,000 points using the voxel-grid filter. An excessive strategy from high sampling interval to low sampling interval was adopted because of differences in the number of point clouds for each raw data, which makes it difficult to obtain a suitable number of point clouds by sampling all the files once. A maximal sampling interval was first set to allow the point cloud to directly down-sample to fewer points. The result was used directly in the subsequent FPS and down-sampled to 15,000 points if higher. In many cases, large sampling intervals make the down-sampled point cloud have less than 15,000 points. In such cases, the sampling interval was gradually reduced until the number of point clouds was more than 15,000 points and then sampled using FPS. The down-sampling results of a plant with 1,383,494 points are shown in **Figure 2**. Notably, although the sampling results obtained herein (**Figure 2**) were similar to those obtained by directly using FPS, the operation efficiency was greatly improved.

### 2.2 Distance field-based segmentation pipeline

In this study, the global morphological structure of corn plant point cloud and the connection relationship of local point cloud were used to segment organs. The end areas of organs (the tip of leaves and the bottom of stems) were sparsely distributed in space, making it easy for positioning. The number of organs on the maize plant and the general position of each organ were determined if the point clouds at the end of these organs were successfully extracted. The rest of the point clouds were divided into corresponding organs based on the connection between the point clouds.

Determining the point cloud at the end of organs is crucial for segmentation. In this study, a special base point was set in 3D space. The Minkowski distance was then calculated from each point of the plant to the base point to form a Minkowski distance field. The location of the base point should be carefully designed to ensure that the Minkowski distance between the point cloud at the end of the organ and the base point is relatively large in the local regions of the plant. Finally, some local regions with large distance values in the distance field were extracted as the end-point clouds of organs.

If **P**_[n]_={**p**_1_⋯**p**_n_} represents the set of plant point clouds to be treated, and p represents a point in the set, the Minkowski distance was defined as the function *F* over the Lebesgue measure on **R** ^3^ as follows:


(1) $$F\left(p_{i}\right)=\|p_{i}-p_{\text{B}}{\|}_{2}{\;}^{\alpha },i=1\cdots n,\;\alpha \geq 1.0,p_{\text{B}}\notin P_{\left[n\right]}$$

where ‖∙‖ represents L2 normal, and α represents a parameter adjusted by the user to increase the contrast of the distance value. **P**_B_ represents the base point. Different base point locations should be selected for different tasks. However, it is necessary to ensure that the distance value of the point cloud at the end of the organ should be large in local areas. Herein, each point cloud was given a distance value to form the plant Minkowski distance field. The position of the point cloud at the end of the organ was determined using the distance value. Locating the end of the point cloud by extracting local maxima may obtain multiple maxima at the end of an organ due to the noise and missing of the point cloud, resulting in over-segmentation. Therefore, the locally high-distance regions in the distance field should be identified. In this study, Quickshift++ was introduced to extract the locally high-distance regions.

Quickshift++ (Jiang et al., 2018) is a new density-based clustering procedure based on the latest development in topological data analysis (Stuetzle and Nugent, 2010; Rinaldo et al., 2012). Quickshift++ consists of two algorithms (1 and 2). Algorithm 1 first works by traversing some k-NN graphs, which encode the level set of k-NN density estimation and form several cluster-cores of the density. Each cluster core represents a data set in which the data are clustered into the same category. The data have a large density value in the local range. Algorithm 2 assigns the remaining data to their appropriate cluster-cores using a hill-climbing procedure based on Quick Shift (Vedaldi and Soatto, 2008).

Quickshift++ was originally used to operate on the k-NN density estimator of data to achieve clustering.In this study, Quickshift++ operates on the Minkowski distance field. We showed that the Minkowski distance function (Formula 1) satisfies the regularity mathematical assumptions of Quickshift++ for input data to theoretically explain why Quickshift++ can perform cluster core recovery and point cloud segmentation in the Minkowski distance field of plant point cloud. The Minkowski distance function has the condition that **p**_B_∉ ***P***_[n]_, and thus it is easy to see that it has a continuous partial derivative and to know that formula (1) is continuous and differentiable. Meanwhile, the function is continuously differentiable and lower bound since it is always greater than 0^1^. Therefore, the function can converge to the local maximum in the gradient direction with local attraction regions^2^. This ensures that the function has cluster cores (locally high-value regions). The points in the attraction regions cluster to these cluster cores along the gradient direction. The corn point cloud is bounded in 3D space. As a result, formula (1) is continuous on the closed interval. Formula (1) is uniform continuity based on the cantor theorem ^3^. This ensures that there is no approximately flat area in the distance field and that Quickshift++ will not get stuck in such a flat area.

The effect of the entire segmentation pipeline is determined based on three parameters: the *K* parameter, the *β* parameter, and *α* parameter. The *K* and the *β* parameters mainly affect the QuickShift++ algorithm. The *K* parameter is used to construct the *k*-NN graph in the QuickShift++ algorithm. The larger the value of *K*, the fewer the number of clusters. The parameter *β*, where 0 < *β* < 1, can be used to adjust the number of cluster-cores in the QuickShift++ algorithm. The smaller the *β* parameter value, the more the cluster-cores are extracted, and the fewer the number of point clouds in each cluster core, causing over-segmentation in the final segmentation category. A larger value of *α* causes a higher sensitivity to the distance change of the local area, yielding more refined segmentation results and more segmented categories. Over-segmentation occurs in such cases. In contrast, the distance contrast of the local areas is not significant when the value of *α* is smaller, yielding fewer segmentation categories.

In this study, DFSP was used to locate point clouds at the end of plant organs and segment the point cloud in the leaf.

### 2.3 Stem segmentation

The median normalized-vectors growth segmentation **(**MNVG) algorithm (Jin et al., 2018b) was used to segment the stem point cloud, starting with a point at the lower end of the stem as the initial seed point. Jin et al. (2018b) obtained the point cloud through human interaction.

In this study, two strategies were used to automatically obtain the initial seed point. The initial seed point was easily obtained when the point cloud of the plant was aligned, making the growth direction consistent with a certain coordinate axis (assuming Z-axis). Several point clouds at the bottom were first selected based on the z-axis coordinates of the point cloud, followed by a calculation of their median point as the seed point. When using Kinect or lidar to obtain point clouds, we can align the obtained point clouds with the coordinate axis by adjusting the position and direction of the sensor.

It is difficult to automatically obtain initial seed points from point clouds that are not aligned. In this study, the plant point cloud obtained by a hand-held scanner was not aligned, and DFSP was used to find the initial seed point.

#### 2.3.1 Extraction of the lowest end of the stem

A base point **p**_B_ was set to make the distance value of the point cloud at the end of the organ belong to the local high-distance regions as far as possible, as follows:


(2) $$p_{\text{B}}=\frac{1}{n}\sum_{i=1}^{n}p_{i\;}\;:\;p_{i}\in P_{\left[n\right]}$$

A small random disturbance was made to the coordinates of **p**_B_ if the coordinates of **p**_B_ were exactly equal to a point in ***P***_[n]_, to ensure that **p**_B_ and the points in ***P***_[n]_ are not equal. The point cloud at the end of plant organs was then extracted *via* DFSP. In this step, DFSP only needs to find the locally high-distance regions of the distance field, indicating that it only needs to execute algorithm 1 in Quickshift++. Furthermore, the K, β, and α parameters in DFSP are represented by *K*_1_, *β*_1_ and *α*_1_ respectively, to distinguish the parameter settings when DFSP is subsequently used.

**Figure 3:**
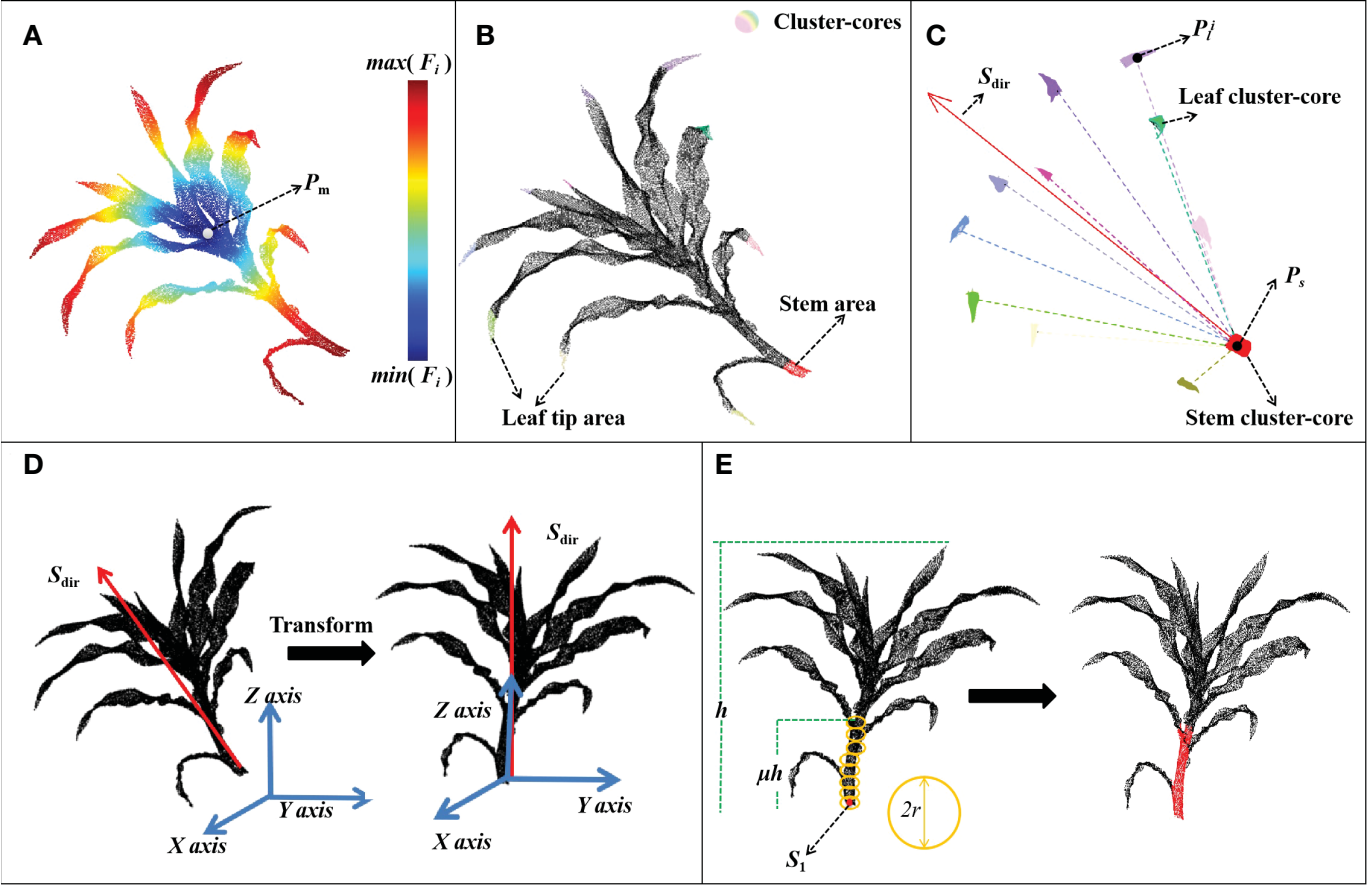
Flow chart of the stem segmentation process. **(A)** A thermodynamic diagram of distance function; **(B)** Stem region extraction based on DFSP; **(C)** Estimation of plant growth direction; **(D)** Plant point cloud alignment; **(E)** Median normalized growth segmentation of the stem point cloud.

The thermodynamic diagram of the distance function is shown in **Figure 3A**. The terminal point clouds of each organ had higher distance values. QuickShift + + was used to extract these organ terminal point clouds, which were then clustered into several cluster cores (**Figure 3B**). In this step, DFSP needs to find the stem area to ensure the correct operation of the subsequent algorithm. DFSP does not need to correctly find all the leaf tip regions. As long as most of the tip regions can be found, the geometric features can be used to identify the stem region.

The local geometric feature of each cluster-core was calculated to determine the stem region. The *m*-th cluster core was set as ***A**_m_*. The set of *k* nearest neighbors of the *i*-th point p*_i_* ^A^_m_ in ***A***_m_ is ***B**_i_* (the number of *k* nearest neighbors was controlled by the variable *K_f_*). Principal component analysis (PCA) was used to calculate the first, second, and third principal component vectors of ***B**_i_* set point cloud, and the corresponding eigenvalues are *λ_1_^i^*, *λ_2_^i^*, and *λ_3_^i^* {*λ_1_^i^* ≥ *λ_2_^i^* ≥ *λ_3_^i^*}. The local geometric feature of *A_m_* was then calculated using formula (3) below:


(3) $$f\left(A_{m}\right)=\frac{1}{M}\sum_{i\in A_{m}}\frac{\lambda_{3}^{i}\left(\lambda_{1}^{i}-\lambda_{2}^{i}\right)}{\lambda_{1}^{i}},$$

where *M* represents the number of point clouds in *A*_m_. *λ_3_^i^* is used to distinguish the planar features of local point clouds. The smaller the λ_3_^i^, the stronger the planar features. (*λ_1_^i^*−*λ_2_^i^*)/*λ_1_^i^* describes the linearity of the local point cloud. The larger this value, the stronger the linearity. Notably, the stem area presents a weak planarity and a strong linear shape compared with the leaf area. Herein, a cluster core with the largest f(*A_m_*) was selected as the stem area, while the rest was the leaf area. Formula validation tests suggested that 98% of the test plant data (420 plants) could correctly get the point cloud at the stem base when *K_f_*, *K*_1_, *β*_1_, *α*_1_ were set at 64, 32, 0.85 and 5, respectively.

#### 2.3.2 Estimation of growth direction

The growth direction of the maize plant was estimated after obtaining the stem and leaf cluster cores (**Figure 3C**). The plant was aligned with the coordinate axis based on the growth direction (**Figure 3D**). Notably, the maize plants presented certain symmetrical characteristics along the stem. The spatial distribution of cluster cores was used to estimate the plant growth direction S_dir_. Supposing that the median point of stem-cluster core was **p**_s_ and the median point of the *i -*th leaf cluster-core was **p** *_l_^i^* the plant growth direction was calculated using formula (4)below:


(4) $${\text{S}}_{\text{dir}}=\text{median}\left\{{\text{p}}_{l}^{i}-{\text{p}}_{s})/{\left\|{\text{p}}_{l}^{i}-{\text{p}}_{s}\right\|}_{2}\right\},i=1\cdots n_{l},$$

where {∙}, ‖∙‖_2_ and n*_l_* represent the median operation, L2 normal, and the number of leaf cluster-cores, respectively.

The coordinates of the point cloud of the plant were transformed after obtaining the growth direction of the plant to ensure that the growth direction **S**_dir_ coincides with the Z axis and **p**_s_ coincides with the original coordinate point. The z-axis was used as the normal vector to project the point cloud onto the plane. PCA assigned the first and second principal component vectors as the x- and y-axes, respectively, of the new coordinate system. The original point cloud coordinates were then transformed into a new plant coordinate system. The coordinates of their z-value were used to judge the height of points in the plant. The height of points increased with greater z-values

#### 2.3.3 Median normalized growth segmentation of the stem

The MNVG algorithm was adopted to iteratively segment the stem point cloud (**Figure 3E**). The coordinate origin was used as the initial seed point **s**_1_ after the plants were aligned, followed by updating the seed point position through multiple iterations and dividing the point cloud around the seed point into the stem point cloud.

Assuming that the algorithm was in the *j-*th iteration and the seed point of this iteration was **s** *_k_*, the specific process of stem segmentation was as follows:

1) Taking **s**_j_ as the center of the sphere, the point set ***A*** within its radius *r* was added to stem point cloud.

2) The growth direction **v**_j_ was calculated using formula (5) below:


(5) $$\begin{array}{c} v_{j}=\text{median}\left\{\left(p_{A}-s_{j}\right)/\|p_{\text{A}}-s_{j}{\|}_{2}\right\},p_{A}\in A\\ v_{j}=\left(v_{j}+v_{j-1}\right)/{\left\|v_{j}+v_{j-1}\right\|}_{2} \end{array}$$

3) The position of the seed point **s**_*j*+1_ in the next iteration was calculated using formula (6) below:


,(6) $$s_{j+1}=s_{j}+rv_{j}$$

4) The end condition of regional growth was then determined when the maximum coordinate of the z-axis of the plant point cloud was *h*. The growth stopped if the z-coordinate of **s**_*j*+1_ was greater than *μh*; otherwise, the next iteration occured, and step 1 was executed.

Notably, **v**_j_ is affected by **v**_j−1_ at each iteration, playing a corrective role in ensuring that the growth direction of the stem is not significantly curved. Herein, **v**_0_ was set as zero vector, while *r* was an adjustable parameter, which was set as the euclidean distance between the farthest two points in the stem cluster core. μ(0 ≤ μ ≤ 1) was a user-adjusted parameter. The larger its value, the longer the segmented stem. The stem segmentation of different plant types of maize was processed by adjusting the value of *μ*. Herein, maize plants before the jointing stage were the main test data, and thus *μ*=0.30 yielded better results.

### 2.4 Leaf segmentation

**Figure 4:**
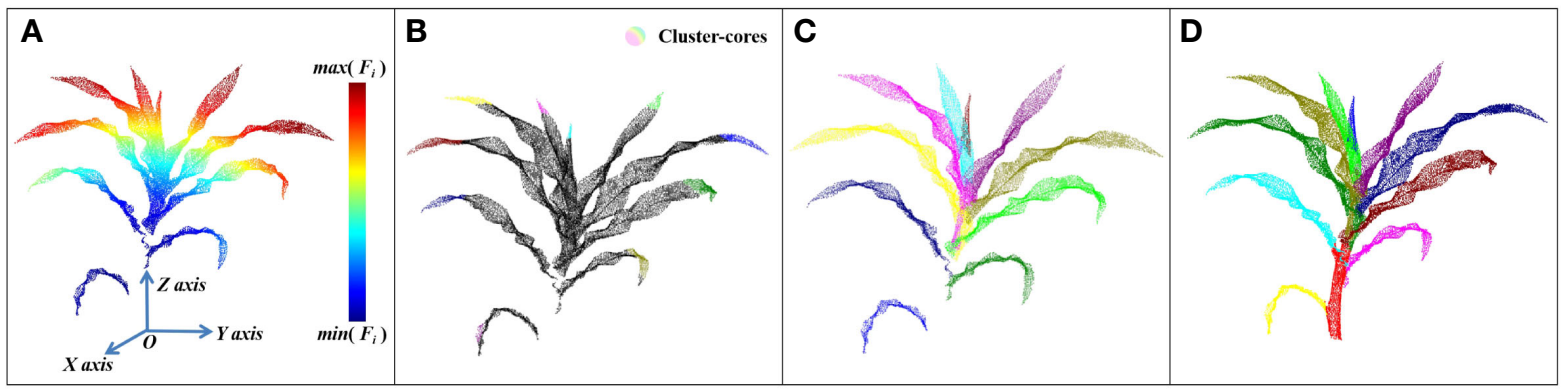
The process of leaf segmentation: **(A)** A thermodynamic diagram of the distance function. **(B)** Extraction results of leaf Cluster-cores. **(C)** Result of leaf instance segmentation. **(D)** Result of stem and leaf segmentation.

The point cloud of maize shoots was spatially divided into several relatively discrete point clouds (excluding the stem) after stem segmentation. However, the end-point clouds of the leaves (adjacent to the stem) were still mixed, making it difficult to segment each leaf. Notably, the tip regions of different leaves of maize were scattered and far away from the lower end of the stem. Therefore, different leaves were separated by extracting the tip point cloud of each leaf to determine the general position and distribution to realize the instance segmentation of the leaves. In the new plant coordinate system, the coordinate of point **p**_s_ in the stem base point cloud was the origin of the coordinate system, and thus **p**_B_ was set as zero vector. The leaf point cloud was segmented using DFSP. DFSP simultaneously executed algorithms 1 and 2 in Quickshift++. In this step, the *K* parameter in QuickShift++ was represented by *K*_2_, while the *β* parameter was represented by *β*_2_ during the segmentation. *K*_2_, *β*_2_, and *α*_2_ were set at 32, 0.85, and 9, respectively, through the test when processing the plant data of 15,000 points to obtain a better leaf segmentation effect. The whole process of leaf segmentation and the final visualization result of stem-leaf segmentation are shown in **Figure 4**. In this study, the new leaves wrapped by each other could be separated, thus enhancing segmentation from the tip.

### 2.5 Statistical analyses

The segmentation results were compared with the ground truth. The precision *P_o_*, recall ;*R_o_*, and F1 score *F_o_* for each organ instance were calculated using formula (7). In the formula, *T_P_* denoted the true positive points, indicating the number of points that were segmented correctly into instance *A; F_N_* represented the false negative points, which were the points originally belonging to instance *A* but wrongly divided into another instance; and *F_P_* represented the number of false positive points in an organ, representing the points of other instances that were wrongly assigned to instance A.


(7) $$\begin{array}{ccc} P_{o}=\frac{T_{P}}{T_{P}+F_{P}} & R_{o}=\frac{T_{P}}{T_{P}+F_{N}} & F_{o}\mathrm{=2}\times \frac{R_{o}{\times P}_{o}}{R_{o}{\mathrm{+P}}_{o}}, \end{array}$$

The precision *P_p_*, recall *R_p_*, and micro-F1 score *F_p_* were also calculated for the individual maize using formula (8). In the formula,

N_o_ denoted the number of organ instances of individual maize.


(8) $$\begin{array}{ccc} P_{p}=\frac{\sum_{1}^{N_{o}}P_{o}}{N_{o}} & R_{p}=\frac{\sum_{1}^{N_{o}}R_{o}}{N_{o}} & F_{p}\mathrm{=2}\times \frac{R_{p}{\times P}_{p}}{R_{p}{\mathrm{+P}}_{p}} \end{array},$$

## 3 Results

### 3.1 Precision/visualization effects

**Figure 5:**
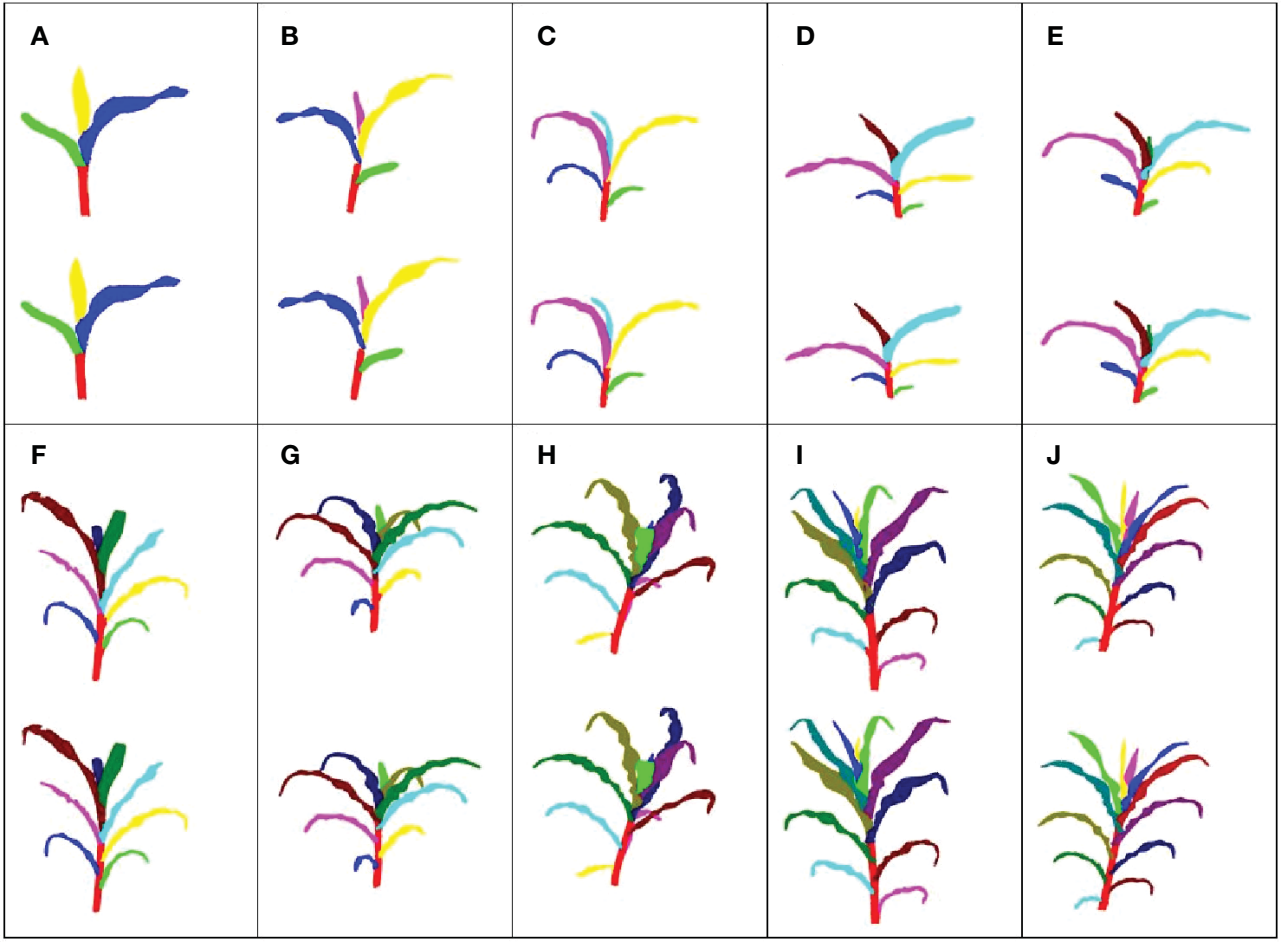
Visualization results of stem and leaf segmentation. The top image in each figure represents the result of our method, while the bottom image represents the ground truth. **(A)** Three leaves; **(B)** Four leaves; **(C)** Five leaves; **(D)** Six leaves; **(E)** Seven leaves; **(F)** Eight leaves; **(G)** Nine leaves; **(H)** Ten leaves; **(I)** Eleven leaves; **(J)** Twelve leaves.

The 420 maize plant point clouds were used to evaluate the segmentation accuracy. Visualization is the most intuitive and effective way to evaluate the accuracy of 3D digital results (Room et al., 1996). The representative segmentation results with different leaf numbers are shown in **Figure 5**. The segmentation results of the leaves had little difference at different leaf positions, indicating that our method was effective for both fully expanded leaves and undeveloped leaves. However, there was a false segmentation at the organ boundary. The numerical accuracy evaluation results for the plant point clouds with different leaf numbers further quantitatively evaluated the segmentation results (**Table 3**). The precision, recall, and F1-score values of each organ instance point cloud were calculated based on the results of the manual segmentation, which were taken as the ground truth.

**Table 3:** The numerical accuracy evaluation results of plant point clouds with different leaf numbers.

	Leaf number	3	4	5	6	7	8	9	10	11	12	AVG
Ours (15000 points)	Precision	0.912	0.903	0.908	0.914	0.924	0.914	0.874	0.892	0.903	0.910	0.905
	Recall	0.903	0.916	0.919	0.919	0.920	0.906	0.867	0.876	0.885	0.882	0.899
	F1-score	0.907	0.909	0.913	0.916	0.921	0.910	0.870	0.884	0.893	0.896	0.902
	Time per plant	1.520 s										
SSA (15000 points)	Precision	0.810	0.877	0.906	0.899	0.864	0.753	0.762	0.741	0.706	0.705	0.802
	Recall	0.810	0.881	0.933	0.925	0.884	0.774	0.780	0.759	0.707	0.678	0.813
	F1-score	0.809	0.879	0.919	0.911	0.873	0.763	0.770	0.749	0.706	0.690	0.807
	Time per plant	57.814 s										
Ours (4096 points)	Precision	0.944	0.898	0.887	0.891	0.891	0.878	0.866	0.834	0.839	0.873	0.880
	Recall	0.924	0.907	0.907	0.899	0.894	0.870	0.856	0.816	0.837	0.838	0.875
	F1-score	0.934	0.902	0.896	0.895	0.892	0.874	0.861	0.824	0.838	0.855	0.877
	Time per plant	0.135 s										
SSA (4096 points)	Precision	0.910	0.868	0.876	0.851	0.805	0.790	0.744	0.651	0.762	0.836	0.809
	Recall	0.917	0.887	0.912	0.885	0.840	0.811	0.765	0.671	0.752	0.791	0.823
	F1-score	0.913	0.877	0.893	0.867	0.822	0.800	0.754	0.660	0.757	0.812	0.816
PointNet++ (4096 points)	Time per plant	14.169 s										
	Precision	0.852	0.891	0.866	0.873	0.854	0.835	0.806	0.820	0.767	0.693	0.826
	Recall	0.835	0.881	0.851	0.858	0.826	0.822	0.797	0.802	0.755	0.690	0.812
	F1-score	0.826	0.878	0.847	0.855	0.827	0.819	0.788	0.799	0.747	0.678	0.806
	Time per plant	0.081s										

### 3.2 Comparison with the existing methods

The developed algorithm was compared with skeleton-based segmentation algorithm (SSA) (Miao et al., 2021a) based on plant data of 15000 point clouds and 4096 point clouds. It was also compared with PointNet++ (Qi et al., 2017) based on plant data of 4096 point clouds. When processing data of 4096 points, *K_f_*, *K*_1_ and *K*_2_ were set at 16, 8 and 8, respectively, while the other parameters were set the same as when processing data of 15000 points. A computer with a Core i7 processor and 32 GB memory was used to test our algorithm and SSA. A computer with a Core i9 processor, 64 GB memory, and RTX 3090Ti GPU was used to train and test PointNet++.

The segmentation accuracy and the average running time of the three algorithms are shown in **Table 3**. The developed segmentation algorithm had good segmentation accuracy and efficiency compared with the other two algorithms. However, its accuracy (F1 score) slightly decreased when dealing with multi-leaf by less than 2%. Moreover, the average running efficiency of the algorithm reached 0.135 s when the algorithm was used to segment plants with 4096 points, which was 100 times faster than SSA and twice slower than PointNet++. However, its average segmentation speed per plant when processing 15000 points was only 1.52 s. Notably, PointNet++ had good accuracy when segmenting plants with four, five, and six leaves (more data). However, its accuracy was significantly reduced when segmenting plants with more than seven leaves. Although the PointNet++ model was limited by its weak generalization ability caused by the data imbalance, it had a good running efficiency. Furthermore, its average processing time was less than 0.1 ms, due to the parallel computing capability of GPU. Although SSA achieved good segmentation accuracy when segmenting point clouds with few leaves, its segmentation accuracy significantly decreased when segmenting plants with more leaves. Obviously, SSA was more limited to the seedling stage. Besides, SSA algorithm had poor running efficiency. Its average running times when processing 4096 points and 15000 points were 14.2 s and 57.8 s, respectively, with most time being spent on skeleton extraction. These results show that the developed segmentation algorithm is effective for maize plant segmentation, especially when there is insufficient data to train a deep-learning network.

**Figure 6:**
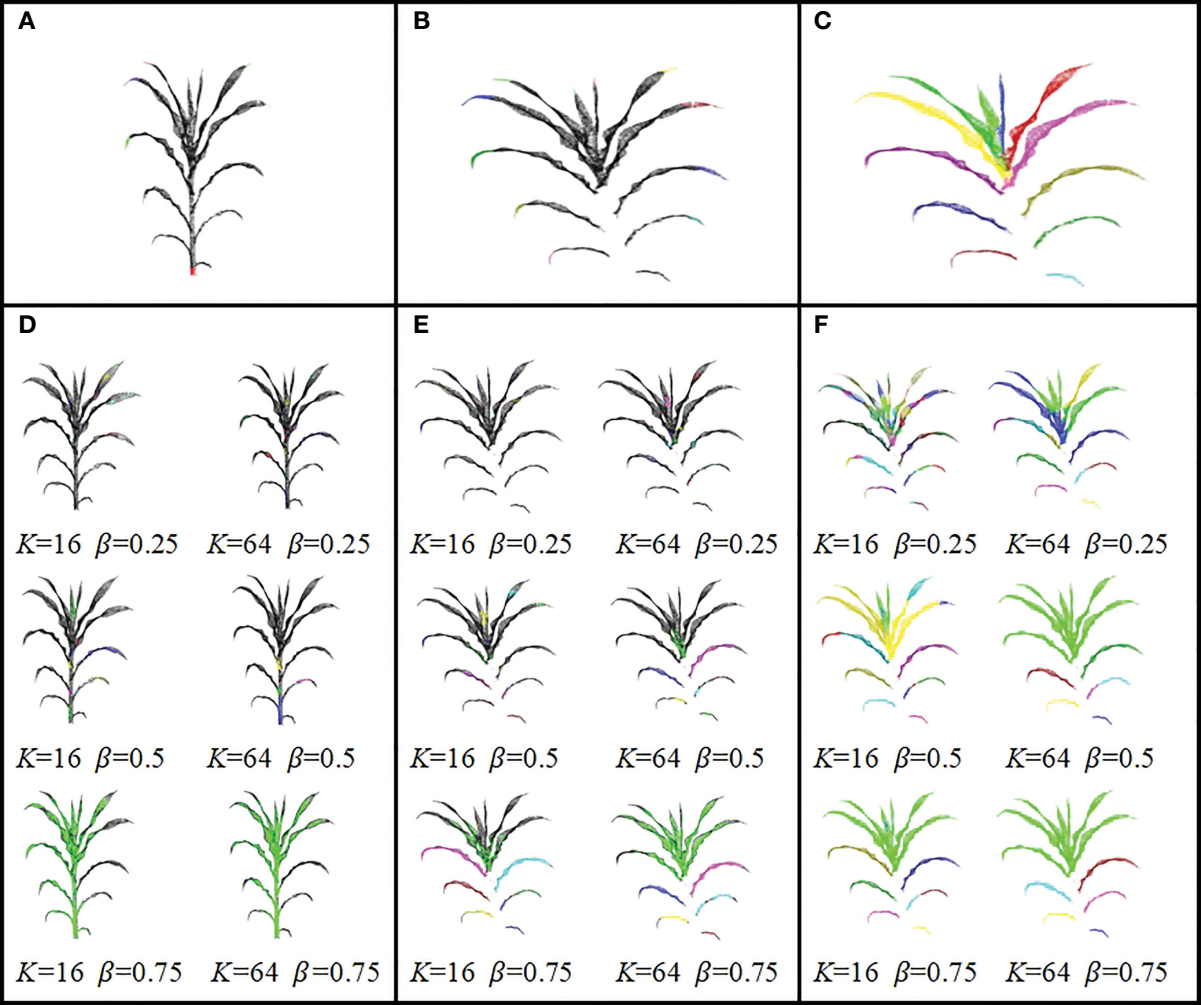
Visualization differences between DFSP and Quickshift++ for organ terminal region extraction and leaf segmentation. The same *K* and *β* parameters are used with DFSP and Quickshift++. **(A)** Organ terminal region extraction using DFSP; **(B)** Leaf tip region extraction using DFSP; **(C)** Leaf segmentation using DFSP. **(D)** Organ end region extraction directly with Quickshift++ under multiple sets of parameters. **(E)** Leaf tip region extraction directly with Quickshift++ under multiple sets of parameters. **(F)** Leaf segmentation directly with Quickshift++ under multiple sets of parameters.

DFSP used two algorithms (1 and 2) in Quickshift++for organ segmentation. However, it should be noted that using Quickshift++directly instead of DFSP cannot perform stem-leaf segmentation. **Figure 6** shows the visualization difference in organ terminal area recognition and leaf segmentation using DFSP and Quickshift++. The reason for this difference is that their inputs were different. DFSP takes the distance field encoding the global structure information of the plant as the input, while Quickshifit++ takes the density field of the point cloud as the input. For organ segmentation tasks, point cloud density is not a useful prior knowledge. This proves that the success of DFSP is not only due to the role of Quickshifit++, but also due to the encoding of plant global features using Minkowski distance fields.

**Figure 7:**
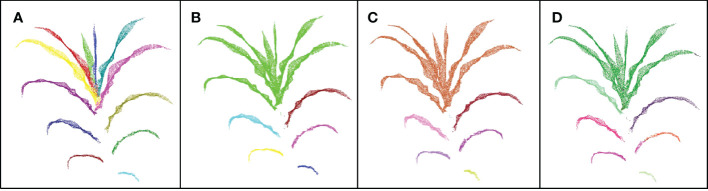
Comparison of leaves segmentation results of DFSP, Quickshift++,DBSCAN clustering method and Euclidean distance-based clustering method. **(A–D)** represents the results of segmentation using the DFSP, Quickshift++, DBSCAN and Euclidean distance-based clustering method, respectively.

Clustering algorithms are often used in point cloud segmentation of maize plants, such as density-based clustering algorithm (Elnashef et al., 2019) and the Eucliden distance-based clustering method (Zermas et al., 2020). We compared the results of our DFSP, two density- based clustering algorithms (DBSCAN and Quickshift++) and Eucliden distance-based clustering algorithm for leaf segmentation (as shown in **Figure 7**). The leaf base parts of new leaves are very close to each other, and the leaf base of a larger leaf covers that of a smaller leaf. None of the three clustering methods can segment such leaves correctly. The fully expanded leaves at the lower part of the plant are far away from each other, and the three clustering methods can successfully segment them. Compared with the three clustering methods, our DFSP is better in the segmentation of new leaves.

## 4 Discussion

### 4.1 Algorithm augmentability

**Figure 8:**
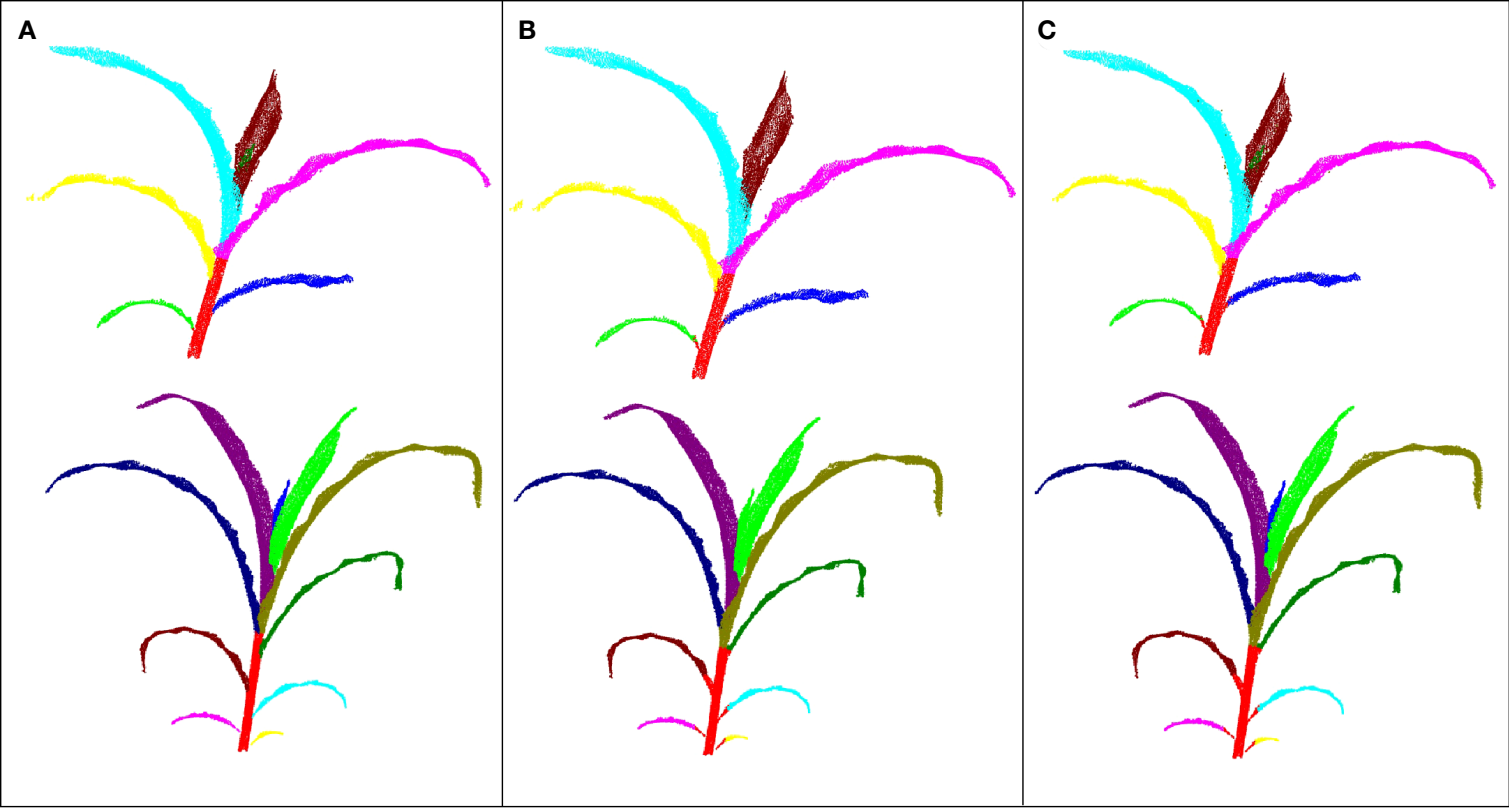
The new leaf segmentation results. **(A)** Ground truth; **(B)** *K*_2_ set at 32; **(C)**. *K*_2_ set at 8.

In this study, all data were processed using similar parameters when validating the algorithm, leading to poor segmentation results for some plants. Notably, the segmentation algorithm was adjustable, providing flexibility for plant segmentation in different plant types and growth periods of maize. A case where the top new leaf was too small, wrapped, and almost stuck to other leaves is shown in **Figure 8**. The general parameters used in leaf segmentation step were invalid for such new leaves. However, small leaves can be extracted to obtain more refined results by reducing the *K*_2_ parameter.

**Figure 9:**
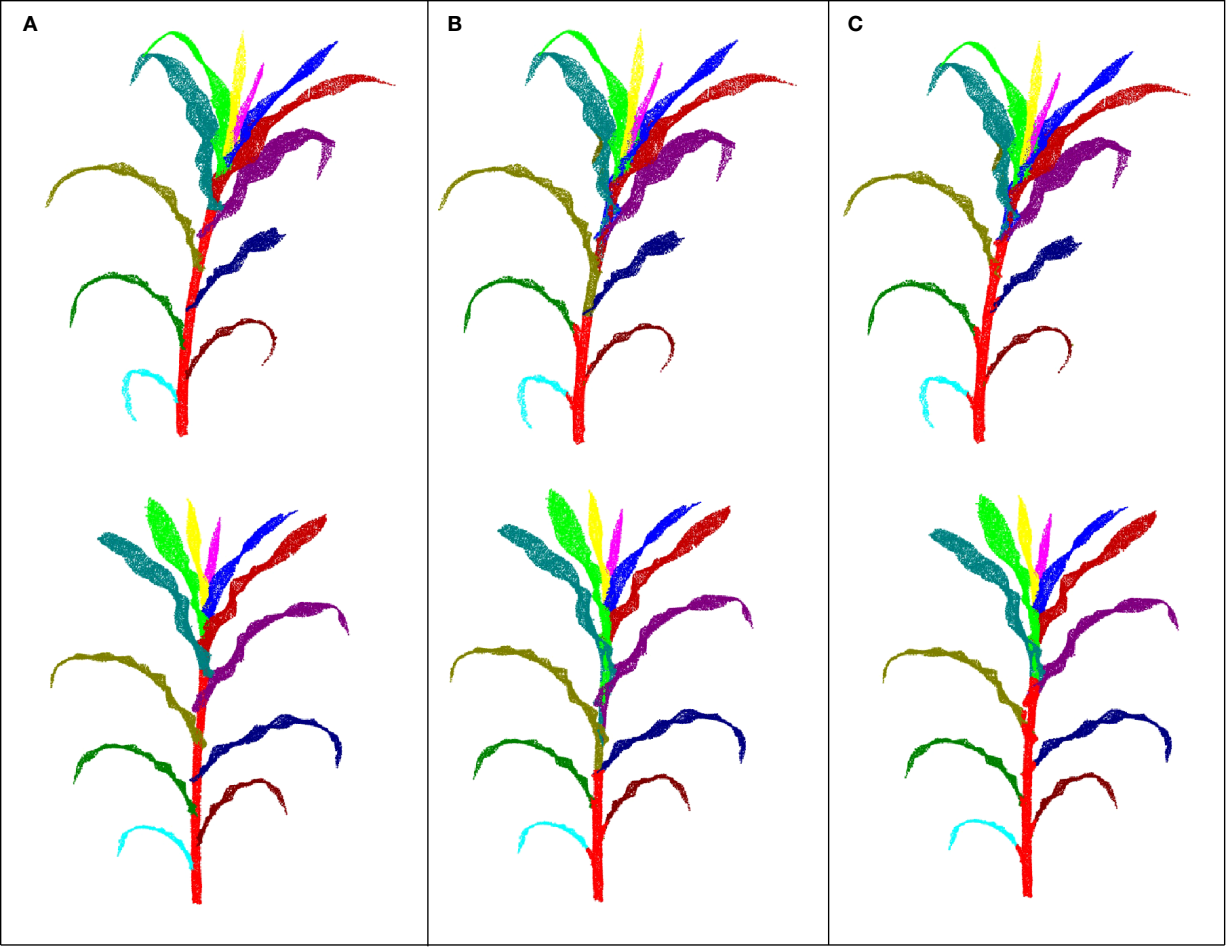
Segmentation results of stems with different *μ* values. **(A)** Ground truth; **(B)** *μ*=0.3; **(C)** *μ*=0.53.

Plants have different heights at different stages. Herein, the data at the seedling stage accounted for the largest percentage, suggesting that the set parameters were more suitable for the point cloud at the seedling stage. Therefore, stem segmentation of higher plants can be under-segmented at *μ*=0.3. However, a better stem segmentation effect can be obtained if *μ* is increased. The effect of different *μ* values when processing point clouds of higher plants is shown in **Figure 9**. Notably, the stem length increased with increasing (*μ* =0.53) value, thus obtaining more refined segmentation results.

**Figure 10:**
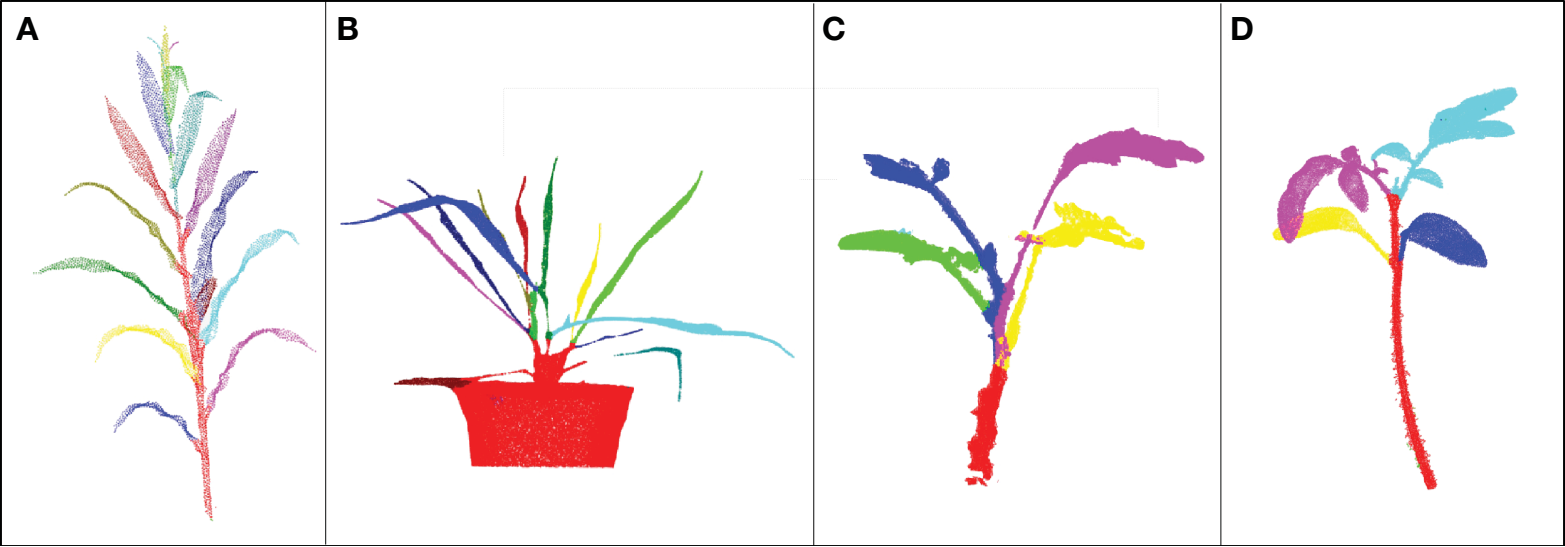
The segmentation effect of the median normalized growth segmentation method on different plants. **(A)** Mature maize **(B)** Potted wheat **(C)** Mimosa **(D)** Eggplant plant.

Besides maize plant segmentation, DFSP has also been used to segment other plants. The segmentation results of mature maize, potted wheat, mimosa, and eggplant using DFSP are shown in **Figure 10**. Notably, the developed method can only apply to stem segmentation of plants with single-branch structures and not to plants with multi-branch structures because it adopts the regional growth method for stem segmentation. However, DFSP has a very good generalization ability and segmentation effect for the segmentation of non-stem organs with different geometric characteristics. For instance, it has a good segmentation effect in mimosa leaves with strong planar characteristics, maize ears with cylindrical characteristics, split tomato leaves, and slender curly wheat leaves, among other characteristics. Therefore, DFSP can be integrated into other segmentation methods and applied to other plant species.

### 4.2 Advantages

The developed method achieved great segmentation accuracy due to the integration of global structural features and local geometric features of the plant. DFSP was used as the core segmentation technology. The Minkowski distance function was used to code the global spatial structure and local connections of the plant. Herein, the sparse distribution of the end positions of various organs of maize in the 3D space was coded into Minkowski distance, which was extracted and recognized with Quickshift++. The point cloud tensor feature, a local geometric feature, in stem recognition was also used. Some few point clouds at the end of organs were first extracted through DFSP to improve the algorithm speed. The stem bottom region could be identified because of the different features on these point clouds. In general, the global and local features of the plant point cloud were integrated *via* DFSP to achieve good segmentation accuracy and avoid introducing complex machine learning technology.

Phenotypic parameters, such as leaf length, width, inclination, stem diameter, and other organ-level parameters, can be calculated through organ-level segmentation. In this study, The algorithm could quickly locate the lower end of the stem and the leaf tip by only one step of cluster-core extraction, thus enabling the extraction of the stem diameter and leaf number parameters without organ level segmentation. Moreover, it enables the calculation of plant height and width after point cloud alignment.

The identification of the lower end of the stem by DFSP also makes the method fully automated, which is crucial for high-throughput phenotype detection. This also ensures that the entire data processing process is non-interactive and less time-consuming. Compared with machine learning and deep learning technologies, the developed method avoids numerous manual annotation work and training processes. Compared with SSA and other methods that use skeleton extraction technology to locate stems, the method also has significant computational efficiency.

Like SSA (Miao et al., 2021b), the developed method can also segment new emerging leaves that are close and wrapped through DFSP. DFSP can be used to identify the leaf tip and segment the blade from tip to base. Jin et al. (2018b) segmented the leaf from the leaf base to leaf tip using the MNVG algorithm. However, this strategy is unsuitable for new emerging leaves (Miao et al., 2021b). Similar to the role of point cloud skeleton in SSA, DFSP can also represent the global topology information of maize plants with significantly reduced time.

A recent work developed Label3DMaize software (Miao et al., 2021a) to label maize point clouds. The software could be used to segment non-stem organs by interactively obtaining the key points of each organ. However, the process of obtaining the key points of each organ is the most time-consuming and labor-intensive step in the whole software. Notably, the developed DFSP segmentation strategy could automatically extract the key area points of each organ, reducing the operational complexity of the entire software.

This study expands the application scope of QuickShift++ in plant point cloud processing from group segmentation (Nelson and Papanikolopoulos, 2021) to single plant segmentation scale. Therefore, the two methods can be easily integrated to develop an organ-level group segmentation tool for the maize. Besides, the relevant existing technologies are mostly implemented by integrating different methods. For example, Jin et al. (2018a) first located the point cloud at the lower end of the stem through the fast RCNN (Girshick, 2015) and then used the regional growth algorithm to achieve single plant segmentation. They finally employed a 3D deep learning network (Jin et al., 2019) to segment the stem and leaf. Ao et al. (2022) first performed stem and leaf semantic recognition through PointCNN (Li et al., 2018) and then used DBSCAN clustering to cluster the stems and leaves into single plants through connection relationships for organ segmentation. DFSP enables group segmentation and organ segmentation through a unified segmentation process, thereby reducing the complexity of developing a maize point cloud segmentation tool.

### 4.3 Limitations and future works

**Figure 11:**
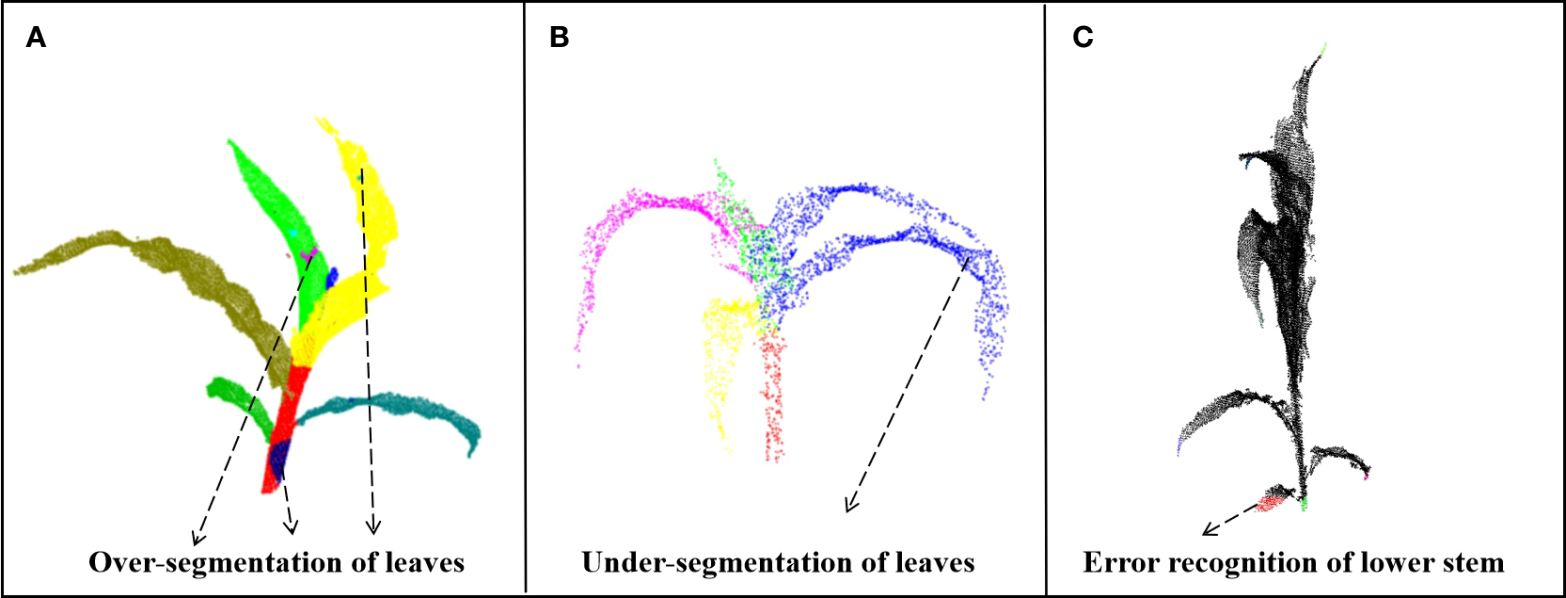
Limitations of the our algorithm. **(A)** Over segmentation of leaves. **(B)** Under segmentation of leaves. **(C)** Error recognition of lower stem.

The proposed method has the following limitations: First, the method used the regional growth method when extracting stems and height when judging how to stop growth, thereby introducing subjectivity despite the increased flexibility. Therefore, future studies should focus on using geometric features to accurately find the junction area between the stem and the top leaf. However, this limitation can be solved using deep learning for semantic segmentation. Second, DFSP is associated with over-segmentation in leaf segmentation (**Figure 11A**). However, when the tip point cloud of one leaf is mixed with another leaf, DFSP will mistakenly segment the two leaves into one instance, resulting in under segmentation (**Figure 11B**). Therefore, post-processing methods should be added in the subsequent works to optimize the segmentation results. Finally, the geometric features (formula 3) used in the identification of stem cluster cores are sensitive to the missing point clouds. The lowest end of the stem can be misidentified if the stem point cloud is seriously missing and its cylindrical feature disappears (**Figure 11C**). Therefore, future studies should introduce more robust geometric features for semantic recognition.

## Data availability statement

The datasets presented in this study can be found in online repositories. The names of the repository/repositories and accession number(s) can be found below: https://github.com/syau-miao/DFSP.

## Author contributions

TM and TX conceived and designed the study. TM designed and implemented the algorithms. DW ran the pipeline on the data sets and analyzed the results. ZS theoretically proved the applicability of Quickshift++ in the distance field. TM, DW and ZS wrote the paper. CZ performed the experiments, acquired the 3D data, and produced the ground truth data. XY run the pointnet++ on the data sets. TY, YZ, and HD improved the approach in some details. All authors contributed to the article and approved the submitted version.

## Footnotes

1 Satisfying assumption 1 of Jiang et al. (2018)

2 Satisfying assumption 2 of Jiang et al. (2018)

3 Satisfying assumption 3 of Jiang et al. (2018)
